# To research (or not) that is the question: ethical issues in research when medical care is disrupted by political action: a case study from Eldoret, Kenya

**DOI:** 10.1136/medethics-2013-101490

**Published:** 2015-10-16

**Authors:** Darlene R House, Irene Marete, Eric M Meslin

**Affiliations:** 1 Emergency Medicine, Indiana University School of Medicine, Indianapolis, Indiana, USA; 2 Academic Model Providing Access to Healthcare (AMPATH), Eldoret, Kenya; 3 Department of Child Health and Paediatrics, Moi University School of Medicine, Eldoret, Kenya; 4 Center for Bioethics, Indiana University, Indianapolis, Indiana, USA

**Keywords:** Research Ethics, Ethics, International Affairs

## Abstract

While considerable attention has been focused on understanding the myriad of ethical analysis in international research in low and middle income countries, new issues always arise that have not been anticipated in guidelines or studied extensively. The disruption of medical care arising as a direct result of political actions, including strikes, postelection violence and related activities, is one such issue that leaves physician-researchers struggling to manage often conflicting professional responsibilities. This paper discusses the ethical conflicts that arise for physician-researchers, particularly when disruption threatens the completion of a study or completion is possible but at the expense of not addressing unmet medical needs of patients. We review three pragmatic strategies and the ethical issues arising from each: not starting research, stopping research that has already started, and continuing research already initiated. We argue that during episodes of medical care disruption, research that has been started can be continued only if the ethical standards imposed at the beginning of the study can continue to be met; however, studies that have been approved but not yet started should not begin until the disruption has ended and ethical standards can again be assured.

## Introduction

Ethical analysis of international research has long focused on issues arising throughout the life-cycle of research, including traditional categories of design, conduct, informed consent, risk-benefit assessment and dissemination of findings.^1–3^ Principles for international collaborative research recommend local institutional review boards to provide independent review, approval and oversight of international research. Informed consent guidelines have tackled questions regarding the conflict between respect for culture and respect for persons, language barriers and cultural scepticism for signing documents.^1^ ^2^ Moreover, discussions regarding appropriate standards of care for research in developing countries have been addressed.^3^ However, there is considerably less ethical analysis undertaken about research challenges in environmental conditions that were not anticipated in international guidelines. One of these is the condition of healthcare service disruption (referred to hereafter as ‘medical’ disruption) where it has a follow-on effect on the capacity to conduct research.

Causes of medical disruption vary from natural disasters to labour actions, from tsunamis to strikes. Whether caused by human action or Mother Nature, all types of medical disruption can impact physician-researcher responsibilities. Unlike disruptions in care caused by tsunamis, earthquakes or tornadoes,^4^ ^5^ we focus here on disruptions caused by the actions of people. These disruptions make it difficult, sometimes impossible to care for patients; and as we emphasise, there is an underappreciated impact on the conduct of research. As resources are stretched, decisions about whether to initiate research or suspend research already underway, and how to balance many conflicting obligations and responsibilities take on a different meaning for host country physician-researchers and for their collaborating colleagues from other countries.

We are particularly mindful of the ethical issues arising from research in diverse settings given our involvement in the two-decade long partnership between Indiana University and Moi University in Eldoret, Kenya called The Academic Model Providing Access to Healthcare (AMPATH).^6^ AMPATH researchers work together to prioritise health problems, determine the value of research proposals, conduct studies, disseminate results and apply the results to improve local health problems.^7^ ^8^ We discuss ethical concerns that arise from our experience with episodes of medical disruption, particularly when disruption threatens the completion of research itself or completion is possible but at the expense of not addressing unmet medical needs of patients.

Some work has already been done to unpack these issues. For example, in recognition of the importance of conducting research in the context of humanitarian emergencies, Médecins Sans Frontières uses a specialised ethics review procedure to handle protocols of this type.^9^ Recently, WHO and the Nigerian Health Ethics Research Committee have developed positions on the use of non-validated treatments in response to the Ebola epidemic.^10^ ^11^ We will discuss ethical issues related to research and clinical care for consideration from our experience during episodes of medical care disruption in Kenya.

## Medical disruption in western Kenya: a case study in research responsibilities

In recent years, the AMPATH programme has experienced several instances of medical disruption. In 2007, postelection violence in Kenya erupted after a disputed presidential election, resulting in the deaths of more than 1200 people, injuring many more, and displacing at least 300 000 people, placing a huge burden on the medical community.^12^ In December 2011, September 2012, December 2013 and January 2015, national physicians’ and nursing strikes took place in Kenya, which involved demands for increased wages and increased allocation of the government's budget towards healthcare.^13^

During the postelection violence, rampant ethnic violence made it unsafe for patients and staff to leave their homes.^14^ Vreeman *et al*^14^ describe some of these experiences: “Families felt trapped…Because it was so life-threatening for them to go on the roads and try to get to clinic.” Lack of public transportation, roadblocks and closure of businesses led to lack of resources and difficulty for staff and patients to get to the hospital. On top of all of these challenges, the horrific terror and stress of seeing neighbours murdered and colleagues’ homes burn provided an overwhelming burden to the community.

On a much lesser scale, the medical strikes also created undue stress for patients. Most patients do not have access to other viable options for healthcare and often felt caught in the middle between governments and healthcare providers. Additionally, these political actions often result in outbreaks of violence. During one of the strikes, police used tear gas throughout the hospital and clinics.^15^ This not only made it more difficult for staff to work, but also created an unsafe environment for already sick patients. Often these strikes result from dangerously low supplies and medications. With the added missing resource of medical staff, meeting patient care needs becomes extremely difficult.

As seen in these events in Kenya, medical disruptions can vary in duration, cause and impact on patient care. Duration of disruptions can vary from minutes or days to months or even years. Knowing that these medical disruptions affect patient care then leads us to consider the impact on research and the ethical responsibilities of physician-researchers. Variations in the types of disruption in care will influence ethical responsibilities of healthcare providers to provide standard of care. As medical disruptions can vary in duration and impact, one can begin to envision several possible scenarios that can result in impact on a healthcare provider's obligation to provide standard of care. For example, a short-term, minor disruption will likely have virtually no impact on the ethical responsibilities of physicians to continue to provide the standard of care of that location. However, a long-term, major disruption would be expected to have significant impact on the ability of physicians to fulfil their ethical responsibilities to provide the standard of care.

While the impact on patient care caused by these political events was well documented, less was known about the impact on research. How might medical disruptions influence physicians as researchers, particularly those that impact patients’ access to care or ability to obtain local standard of care? These episodes of medical disruption cause significant pause for physician-researchers regarding how to proceed with research.

## Conflict in research responsibility

In international collaborative research, physician-researchers often have to balance clinical duties and research responsibilities. In many low income and middle income countries, the environment in which healthcare is delivered and research is conducted is often one and the same. This is often the case in resource-constrained settings where shortages of healthcare providers or limited access to care leave physician-researchers to serve in both roles, making these ethical responsibilities difficult to separate.^16^ In times of medical care disruption, balancing these roles appropriately can become very difficult. With local physician shortages, the physician-researcher often may be the only physician a patient will see. With demanding clinical needs and responsibilities, one can expect that during circumstances of medical care disruption host and visiting physician-researchers struggle with how to proceed with research. Physician-researchers who serve in clinical and research roles may feel pulled to more clinical responsibilities to meet those needs, making it difficult to fulfil research responsibilities. Physician-researchers with primary research responsibilities may also be pushed to take on clinical duties to meet needs of patients who now have limited access to care.

The risks to research that we consider balancing in this discussion primarily surround the duty to care, which is especially strong where relationships between patient-participants and physician-researchers already exist. Disruptions often result in limited resources and personnel, which can lead to difficulty with resource prioritisation for care versus research as well as difficulty adhering to protocols. Additionally, in light of political disruptions affecting healthcare, patients may be particularly prone to issues of trust and exploitation as already influenced by political agendas that must be taken into consideration when asking to participate in research. It is important for these international collaborative research partnerships to avoid exploitation in these difficult situations by working in solidarity, engaging community members and fostering development of these healthcare systems when making decisions regarding continuation of research.^17^ With this in mind, we review three general arguments for not starting research, stopping research that has already started and continuing research.

## Argument for not starting research

It may seem intuitively obvious that during times when political unrest affects access to medical care clinical research should not be started. These political disruptions create risk to the public generally and can prevent timely delivery of resources, limited access to trained personnel, and increased stresses for patients and clinicians.^8^ When disruptions turn dangerous, it is easy to see how the first order of business is to care for patients in need, and not research subjects in studies.

Additionally, the ethical ground is less secure for research participants. It is an accepted principle of research ethics that research participants should not be exposed to unreasonable risks, especially when there is no prospect of benefit. Typically, this balance is focused on risk and potential benefit within the study itself, but it would be naïve to exclude the risks to subjects from political disruption since all research participants would be exposed to them. In a high-risk environment where medical services are needed and otherwise unavailable, there is an increased risk to autonomy as patients may be more susceptible to joining research as their only way to access care despite being well informed.

Moreover, disruption of service dramatically increases the likelihood of incomplete or compromised data, loss of controlled conditions and introduction of confounding variables into the final analysis, diminishing the possibility of answering the proposed research question. Since the benefits of research are often to science and society as a whole rather than to the individual, benefit from the study is also diminished. Also, since no researcher-participant relationship has formed, there are no expectations of participants for beginning or continuation. Therefore, if the decision to start a new project increases the likelihood that current patients will be worse off because resources may be diverted from their care, then the ethical justification for starting research is difficult to make. While research has the potential to benefit the health of populations, the risks overall are too high to start research during medical care disruption. The prudent course is to wait until after resolution of these episodes when ethical standards can be met, the safety of patients and research subjects assured, and the likelihood of completing a study is maximised.

## Argument for stopping research already begun

It may be ethically more expedient not to start a study but is there an ethically relevant distinction within clinical research ethics between stopping something underway and not starting? We think there is. At a minimum, to stop a study already underway will involve the dashing of certain expectations and commitments that not starting does not. Investigators expect studies to be seen to their conclusions, research ethics committees give their approval to studies expecting them to be completed, funding bodies support finished rather than unfinished studies, and patients entering a study have made certain commitments to themselves, science, and others. Of course, under some circumstances studies should be stopped for legitimate reasons. Studies determined to have perturbed equipoise sufficiently should be stopped by Data Safety and Monitoring Boards, as should studies that have been found to be non-compliant with federal regulations.^18^

When relevant country-specific guidelines and regulations are ambiguous or silent on this issue, researchers face a difficult decision and may seek input from review boards or other experts in such matters.^19^ When the disruption is from a natural disaster, the same public safety reasons that support not starting a study would also militate against continuing. When research disruption is from a ‘political’ situation, similar considerations apply: where the disruption creates an immediate public health risk, as occurred during the 2007 Kenyan election violence, studies underway could sensibly be stopped. But what about disruptions caused by peaceful labour action? Here the argument for continuing a study is stronger where physician-researchers may still be able to carry out their duties, making continuing research possible as compared with violent protests. However, where the disruption targets healthcare facilities, preventing the very clinicians who are also researchers from working, the capacity to continue studies is substantially lessened.

Physician-researchers have ethical obligations to ensure care for their patients.^20^ ^21^ This obligation applies whether one is in a trauma centre in Boston or an outpatient clinic in Burnt Forest, Kenya. The Declaration of Helsinki makes several statements on this issue in its introduction, including ‘the duty to promote and safeguard the health of patients’ along with ‘the well-being of the individual research subject must take precedence over all other interests’.^22^ Therefore, if clinical care is not being provided and a trade-off in providing care over research is required, it is permissible to stop research until ethical standards can once again be met. This responsibility to ensure care is made particularly strong where research has already begun and a relationship already exists between the patient and physician-researcher. In the case of a study that holds out the prospect of benefit to subjects but whose continuation could only occur by withholding care to other patients, again we would favour stopping the study. Once the disruption has ended and ethical standards can again be met, research should be restarted as quickly and feasibly as possible.

Given the nature of international collaborative partnerships, consultation with local communities, collaborators and participants is essential to making the decision to stop research. The goal of such partnerships is to ensure fair benefits to local physicians and communities and cannot be ignored when considering this difficult decision.^17^ If communities and participants feel that continuation is undesirable and discussion continues to reveal concerns of increased risk and uncertainty regarding value of the research during disruptions in medical care, discontinuing the research for this time would be included in the physician-researcher's responsibilities to these relationships. Commitment and trust will continue to build relationships and provide the groundwork for future research.

Additionally, continuing to conduct research during periods of medical care disruption not only affects patients, but also affects research. Lack of resources, medications and other stresses often lead to difficulties in following research protocols. When patients are not receiving optimal care, the validity of research results is likely affected. This may place patients at undue risk if the results from the study then are not useful or generalisable. In this scenario when political events have disrupted care and research in a way that undermines the scientific validity of results, researchers must exercise caution when considering to continue providing study medicines or interventions as it is difficult to assess whether research is benefiting subjects and it is potentially harmful to continue providing these medicines when medical monitoring is not possible. As research has the potential to be negatively affected ethically and scientifically during times of medical care disruption, physician-researchers must strongly consider the effects on their research and whether continuation is worth pursuing while an already resource-limited medical system is overwhelmed. Many research questions can and have been answered retrospectively during these events, but careful consideration regarding necessity, feasibility and risk-benefit ratio must be given to continuing studies when medical care is disrupted.^14^

When a study must be stopped because of a political disruption, discussion of post-trial obligations to subjects takes on new meaning since benefits to subjects are difficult to assess, and thus researchers should be cautious when considering to continue providing study medicines or interventions as mentioned above. Researchers should continue to keep study subjects informed and monitor their progress to the extent possible.^18^ ^22^

## Argument for continuing research

Conducting research is one of the most important means to improve health.^23^ So, continuing to focus on research that has the potential to improve the health of many more patients can be defended on ethical and pragmatic grounds. The sooner a study is completed, the sooner any results can be used to inform care. Indeed, for some studies, discontinuing a study exposes participants to unnecessary risk with no prospect of benefit to themselves. Continuing research honours the commitment that researchers have made to research participants; breaking these commitments might further damage confidence and trust towards medical research.^24^ Additionally, community engagement identifies and prioritises research with particular importance to their society so continuation would continue to promote benefits for their local community. If communities and participants believe this research was important to continue and did not pose any increased risk, continuation would be important to promote trust between partnerships involved. Also, whether research is clearly distinguished from clinical care or seen as integrated with care, the impact of a disruption on one should not in principle disrupt the other.

Conditions exist under which the study could be continued. Research should continue if physician-researchers and community members agree that continuation of research was the appropriate way to proceed, assuming critical evaluation of each party's reasons to proceed. The ethics review committee that approved the study is then made aware of the disruption of care, and confirms that the disruption will not adversely affect the conditions of approval placed on the study, including consent procedures, opportunities for withdrawal and availability of study personnel. A study already approved by an ethics committee that continues to meet ethical standards can continue to be conducted.

These points make the case for *permitting* a study to continue under conditions of politically caused medical disruption. However, physician-researchers must carefully consider the availability of existing resources, safety of human subjects and reliability of results. For example, they may also have to determine how continuing a study might prevent a prior agreement to provide care from being fulfilled.^25^ Given the increased risks and vulnerability of the populations, studies should not be started unless they can be assured of being completed.

## Additional considerations

When considering whether to start, stop or continue research, the type of research and research aims will likely influence this decision. One may consider observational, low-risk studies less burdensome and therefore, more permissible; however, the aims of the study may be of little comparable importance to the society in distress that it may seem unimportant to begin or continue until the situation has resolved or stabilised. On the other hand, while interventional studies are quite burdensome, they may be providing needed treatment for patients. Additionally, if the aims of the study are of particular importance during times of medical care disruption such as studies that address how to optimise healthcare during times of disruption, it may shift the balance of decision making in favour of starting or continuing research. It will be important to consider the burden of these studies on an already burdened population, engage local communities regarding importance and expectations, and ensure ethical standards are met.

## Conclusion

Conducting research in developing countries offers unique challenges, especially during unforeseen and stressful situations involving medical care disruption from political action. While working in Kenya, these events have affected research and caused physician-researchers to wonder how to proceed. With increasing research in developing countries, more discussion needs to occur regarding physician-researcher obligations during times of medical care disruption. Whether circumstances include political unrest, violence, physician strikes, etc, guidelines are needed to help physician-researchers prioritise ethical obligations during already difficult, stressful situations. We offer the following recommendations regarding how to proceed with research during medical care disruption in resource-constrained settings.

Overall, research is extremely important to improve patient care and should be encouraged, especially in resource-limited countries. Research should always be conducted according to recognised ethical standards for protection of human subjects and promotion of scientific integrity with a specific focus on a physician-researcher's duty to provide care to patients. We believe there is an ethical distinction between starting and continuing research. During periods of medical care disruption, research that has already been started should continue when possible. When research cannot meet these ethical standards, it should not be started or, if already underway, should be ethically stopped. Therefore, if the conditions in a country prevent or seriously impede a researcher's ability to meet ethical standards required of the protocol, then a study that is underway should be stopped or wound down in a manner consistent with patient safety until ethical standards can be assured. If the cause of the disruption ends, and it is possible to restart a previously stopped study it should be restarted as quickly and feasibly as possible. In countries where care disruption may be reasonably foreseen, either from natural or human-caused factors, efforts should be made to anticipate such situations and to include plans in the protocol and consent form so that the institutional review board is aware of the possibility as it undertakes its review that a study might stop, and patients who are about to be enrolled would know of this additional disclosure and risk of their participation when they are reviewing consent materials. Studies that have been approved but not yet started should not begin until the disruption has ended and ethical standards can again be assured.
